# Neural Processing of Odors with Different Well-Being Associations—Findings from Two Consecutive Neuroimaging Studies

**DOI:** 10.3390/brainsci13040576

**Published:** 2023-03-29

**Authors:** Akshita Joshi, Henriette Hornstein, Divesh Thaploo, Vanda Faria, Jonathan Warr, Thomas Hummel

**Affiliations:** 1 Smell & Taste Clinic, Department of Otorhinolaryngology, Faculty of Medicine Carl Gustav Carus, Technische Universität Dresden, Fetscherstraße 74, 01307 Dresden, Germany; 2 Department of Psychology, Uppsala University, 75185 Uppsala, Sweden; 3 Brain and Eye Pain Imaging Lab, Pain and Affective Neuroscience Center, Department of Anesthesiology, Critical Care and Pain Medicine, Boston Children’s Hospital, Harvard Medical School, Boston, MA 02115, USA; 4 Takasago, 75017 Paris, France

**Keywords:** well-being, olfaction, fMRI

## Abstract

Much is known about the effect of odors on mood, cognition and behavior, but little is known about the relationship between odors and well-being. We investigated the neural processing of odors with different degrees of association with well-being (WB) through two large independent datasets. The study encompassed pre-testing and fMRI. During pre-testing, 100 and 80 (studies 1 and 2) young, healthy subjects participated, rating intensity, valence, and WB association for 14 (study 1) and 8 (study 2) different odors. Pre-testing resulted in the selection of two odors with high WB association (WB-associated) and two odors with lower WB association (neutral odors) for each study. Odors were delivered intranasally to the subjects who underwent fMRI scanning (44 and 41 subjects, respectively, for studies 1 and 2). We assessed brain activity for subjects when they experienced WB-associated versus neutral odors. In study 1, WB-associated odors showed increased activation in the right angular gyrus whereas in study 2, increased activity in the left angular gyrus existed, together with increased activity in the anterior cingulate cortex and posterior orbitofrontal cortex. The increased activity of higher-order cognitive and emotional regions during the processing of WB-associated odors in the two independent studies suggests a role of odors in influencing individual well-being. Moreover, the consistent activation of the angular gyrus might suggest its key role in shifting attention toward relevant emotional stimuli.

## 1. Introduction

Well-being (WB) has been defined as a healthy state of mind, when an individual is able to cope with the normal stress of life, is able to work productively, is confident and is capable of contributing to their community [1]. More than just the absence of disease, a healthy state is also defined as a state of complete physical, mental and social WB [2].

Odors can have a strong impact on mood states. For example, unpleasant odors have been shown to affect mood negatively and increase pain perception [3,4], whereas pleasant odors seem to improve mood, elevate work productivity, and increase calmness [5,6], alertness and attentiveness [7]. The connection between mood and olfaction is neurofunctionally related to an overlap between olfaction and primary “emotional areas” such as the amygdala, insula, orbitofrontal cortex (OFC) and cingulate cortex [8,9]. This intricate relation becomes even more salient in the presence of smell loss. People with smell dysfunctions often report a negative impact on mental and emotional health, which in turn affects WB [10,11]. Patients with severe olfactory dysfunction have been shown to have significantly lower WB [12].

On the other hand, odor perception is context-dependent and can be modulated by cognitive variables such as mental models of how odors relate to the environment and associative learning based on prior experience or situational contexts of odorous experiences [13,14].

Neuroimaging techniques have been used to investigate neuro-functional and neuroanatomical correlations between mood, cognition and olfaction. Odors can modulate mood and behavior. When perceiving odors, the insular cortex, part of the secondary olfactory cortex, was found to be associated with improved emotional awareness or assessment of emotional status [15,16]. Another region of the secondary olfactory cortex, the OFC, is also activated when odors influence cognitive, social and emotional processing [17]. Activation of the OFC is also associated with the pleasantness of stimuli and in part is involved in processing the reward value and affective aspects of olfactory stimuli [18].

Overall, previous literature reports have shown the impact of odors on mood and behavior separately but it is unclear how odors affect WB, which amalgamates physical, mental and social domains. Does the neural processing of odors associated with WB differ from the processing of odors that show little association with WB?

To our knowledge, there are no imaging studies evaluating the central nervous processing of odors and their associations with WB. We performed two consecutive studies in two distinct populations to understand how humans process odors with different WB valence. Whilst study 1 had an exploratory approach, study 2 was focused on replicating the activation patterns seen in study 1. We hypothesized that odors associated with WB to greater or lesser degrees would differently activate regions involved in higher order cognitive, reward and emotional processing. To avoid confusion in the manuscript, we will be using the term “neutral odors” for odors that have little association with WB.

## 2. Methods

### 2.1. Study Design and Participants

Both studies were approved by the Ethics Committee at TU Dresden and were conducted according to the Declaration of Helsinki. All participants provided written informed consent. Participants were recruited with the help of flyers displayed across the university campus.

We recruited healthy participants with the exclusion criteria of olfactory dysfunction, clinically assessed depression, physical or mental illness, a regular intake of medication (apart from contraceptives), alcohol consumption on a daily basis, and a history of any neurological diseases affecting the sense of smell. The health status of participants was ascertained using their detailed medical history [19]. Participants self-assessed their olfactory ability on a 6-point scale (0 = “no sense of smell”; 1 = “very bad”; 2 = “bad”, 3 = “normal”; 4 = “good”, and 5 = “very good”). Participants who self-assessed as normosmic or better were recruited. Normal olfactory sensitivity was ascertained using a 16-item odor identification test (with a score ranging from 0 to 16) from the “Sniffin’ Sticks” olfactory test. This test includes a forced-choice paradigm in which subjects have to identify 16 odors at supra-threshold concentrations using flash cards with four descriptors each [20]. The mean identification scores of subjects who underwent fMRI scanning were 13.6 ± 1.1 (study 1) and 13.3 ± 1.4 (study 2), respectively. All tests were performed in well-ventilated rooms. The purpose of pre-testing was to select two odors with high WB association and two neutral odors that were then presented intranasally to the subjects during the fMRI scanning session. Odors were selected from different categories: nature, sweets, fruits, flowers, laundry, etc. Subjects subjectively rated the odors according to their preferences for their association with well-being. 

### 2.2. Study 1

During pre-testing, one hundred healthy participants (44 women and 56 men) of a mean age of 26 years were recruited. Subjects filled in a general WB questionnaire describing their general state of WB. A German version (Fragebögen zur Beurteilung des Wohlbefindens) and an English-translated version of this questionnaire were used according to the language subjects were most familiar with. This test includes psychological, physical and mental subscales [21] (Wydra, 2003). It has 42 questions such as I am physically resilient; I feel physically balanced (physical WB); I am in a good mood; in the evenings, I feel pleasantly tired; I have everything under control (mental WB); I would like to help other people; I feel loved; I have lots of friends (social WB). Marking was carried out using a 5-point scale (from “yes exactly” (5 points) to “not at all true” (0 points)).

For the pre-testing, the 14 odors (all odors were provided by Takasago, Paris, France) presented were lavender, apple strudel, forest, musk flower, peppermint, lilac, orange, rose, vanilla, chocolate, coffee, grass, cedarwood oil and leather. Both during pre-testing and MRI scanning, subjects rated the odors for their intensity, valence and WB associations. From the 14 odors presented during pre-testing, “musk flower” (mean (M) = 7.45, standard deviation (SD) = 1.9) and “orange” (M = 7.04, SD = 1.65) were rated as odors with the highest WB association, whereas “coffee” (M = 4.97, SD = 2.17) and “grass” (M = 4.29, SD = 2.10) were rated as neutral odors. From 100 subjects who participated in pre-testing, 44 subjects were recruited for fMRI scanning. These 44 subjects (23 of whom were men; mean age = 25, SD = 3 years) exhibited a similar pattern of rating musk flower and orange as most associated with WB and coffee and grass as least WB-associated. 

We performed ANOVA with repeated measures with a Greenhouse–Geisser correction to obtain the sequence of the odor ratings. The mean scores for the valence of the four odors were statistically significantly different (F (2.36, 101.79) = 37.1, *p* < 0.001). A post hoc analysis with a Bonferroni adjustment revealed that the valence ratings significantly differed for coffee compared to musk flower (−3.65 (95% CI, −4.88 to 2.43), *p* < 0.001) and orange (−3.20 (95% CI, −4.39 to 2.01), *p* < 0.001), and also for grass compared to musk flower (−3.63 (95% CI, −4.81 to −2.46)) and orange (−3.18 (95% CI, −4.68 to −1.68)). The order of valence ratings was musk flower > orange >> coffee > grass.

In terms of the order of the WB ratings, the WB ratings differed significantly for the four odors (F (2.65, 114.01) = 28.6, *p* < 0.001). The WB ratings decreased for coffee compared to orange (−2.07 (95% CI, −3.12 to −1.012), *p* < 0.001) and musk flower (−2.47 (95% CI, −3.61 to −1.34), *p* < 0.001), and also significantly decreased for grass compared to orange (−2.75 (95% CI, −3.99 to −1.50), *p* < 0.001) and musk flower (−3.16 [95% CI, −4.26 to −2.05], *p* < 0.001). Order of the WB ratings was musk flower > orange >> grass > coffee.

### 2.3. Study 2

Eighty healthy participants (42 women and 38 men) of a mean age of 27 years were recruited for the pre-testing. The study was divided into two parts. During pre-testing, we included more questionnaires for subjects to have a better understanding of WB and to understand each individual’s WB, happiness and mental state better. We included the WHO-5 well-being scale [22], subjective happiness scale [23], general WB questionnaire [21], Warwick-Edinburg Mental WB scale [24], and WB questionnaire-12 [25]. In this study, subjects rated eight different odors for their intensity, valence and association with WB. The odors were chocolate cake, white fresh aldehydic soap or ‘white soap’, grass, forest, vanilla, lavender-scented ‘sheets’, rose flower, and clean linen (provided by Takasago, Paris, France). From these eight odors, based on their WB ratings, the grass (3.49 ± 2.41) and sheets (3.37 ± 1.84) odors were selected as neutral odors with minimum WB scores and vanilla (6.98 ± 2.13) and white soap (6.46 ± 2.10) were selected as WB-associated odors with maximum scores. In addition to the ratings of intensity, pleasantness and WB associations, we also asked the subjects to arrange the odors in a sequence from their lowest to highest WB association. After an interval of 10 min, the subjects were asked again to go through the sequence they made and confirm it. In this way, the subjects were confident with the selection of odors. Additionally, for study 2, the four selected odors (2 of which were WB associated and 2 of which were neutral odors) were given to the subjects in prefilled soft nose clips to wear for two hours once; that is, one odor was to be worn per day, each for 2 h. They were recommended to wear the clips at home pre-dinner and to avoid eating or drinking 30 min prior to wearing the clip. This gave more exposure to the odors, which was a limitation reported by the participants from study 1. As WB can be better assessed over a period rather than at an instance, this longer exposure period allowed the subjects to more clearly decide if the odors improved their WB or not.

From 80 subjects we recruited 41 subjects for fMRI scanning. These subjects had similar selection of neutral and WB-associated odors. As per the ratings there was no significant difference in odor intensity (F (2.36, 94.59) = 4, *p* = 0.69). When using an ANOVA with repeated measures with a Greenhouse–Geisser correction, the mean scores for the valence of the four odors were statistically significantly different (F (2.71, 108.39) = 37.7, *p* < 0.001). The post hoc analysis with a Bonferroni adjustment revealed that valence ratings significantly decreased for grass compared to vanilla (−3.43 (95% CI, −4.64 to 2.23), *p* < 0.001) and white soap (−3.29 (95% CI, −4.56 to 2.02) *p* < 0.001), and also decreased for sheets compared to vanilla (−3.51 (95% CI, −4.85 to −2.17)) and white soap (−3.36 (95% CI, −4.49 to −2.24)). The order of the valence ratings was vanilla > white soap >> sheets > grass. The WB ratings varied significantly (F (2.82, 112.77) = 46.55, *p* < 0.001). The WB ratings decreased for sheets compared to vanilla (−3.61 (95% CI, −4.83 to −2.38), *p* < 0.001) and white soap (−3.09 (95% CI, −4.10 to −2.09), *p* < 0.001), and also significantly decreased for grass compared to vanilla (−3.48 (95% CI, −4.70 to −2.27), *p* < 0.001) and white soap (−2.97 (95% CI, −3.98 to −1.96), *p* < 0.001). The order of the WB ratings was vanilla > white soap >> grass > sheets.

### 2.4. Odor Presentation in the Scanner

During the fMRI measurements, the selected odors were delivered birhinally using Teflon tubing connected to a portable computer-controlled olfactometer [26] in a room next to the scanner room. Odorous stimuli (undiluted) were embedded in a 2 L/min constant airflow. Each subject underwent four sessions with one type of odor being presented in each session. The order of sessions was randomized among the subjects.

**Figure 1:**
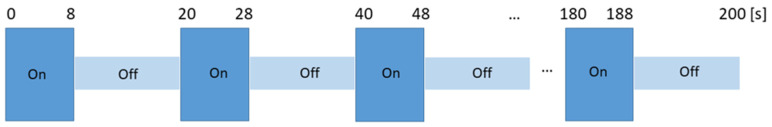
Experimental design for odor presentation. ON session corresponds to the “odor session” where an odor was released for 8 s and OFF session corresponds to the “unscented air session” lasting 12 s. ON-OFF sessions were repeated 10 times. Four sessions were conducted per subject, each lasting 200 s (s).

The odors were presented in a block design format (Figure 1). The block design had 10 alternative blocks of ON/odorous stimuli (8 s) and OFF/unscented air (12 s) each. In the MR scanner, after each odor was presented, subjects rated the intensity (on a 10-point numerical scale: 0–10), pleasantness (on a 5-point Likert scale: −5 to +5) and the WB-associated with the odors (on a 10-point numerical scale: 0–10). Subjects communicated through the scanner intercom system.

### 2.5. fMRI Acquisition

Data acquisition was performed on a 3T MRI scanner (“Trio” model, Siemens Medical Systems, Erlangen, Germany) using a 32-channel head coil. A total of 248 functional images were collected using a T2 single-shot echo-planar imaging (EPI) sequence: TR  =  869 ms, TE  =  38 ms, 58° flip angle, no interslice gap, and 210 × 210 mm field of view. A high-resolution structural T1 image was acquired using a 3D magnetization prepared gradient rapid acquisition gradient echo (MPRAGE) sequence (TR  =  2000 ms, TE  =  1.95 ms, 256 × 256 mm^2^ field of view, and voxel size 1 × 1 × 1 mm^3^). In study 2, we changed the TR to round figures of 1000 ms to match the number of scans with the TR. All other parameters were the same for both parts.

### 2.6. MRI Data Preprocessing and GLM Analysis

The task-driven general linear model approach (GLM) using Statistical Parametric Mapping (SPM) software version 12 (version 12; http://www.fil.ion.ucl.ac.uk/spm/ accessed on 15 February 2021) [27], which is a MATLAB R2018b-based software (The Mathworks Inc., Natick, MA, USA) was used for the analysis of functional MRI data, (Welcome Trust Centre for Neuroimaging, London, UK). Preprocessing steps in SPM12 were set at default. 

The steps of pre-processing included realignment, unwarping, co-registration, segmentation, smoothing and normalization. First level analysis was conducted with the standard hemodynamic response function in SPM. For each individual, contrast images for “ON > OFF” were generated. Activations significant at uncorrected *p* value of < 0.001 with a cluster size of (k) > 10 voxels were reported. For clusters with multiple peaks, the one with the highest *t*-value was chosen. 

Ten blocks of ON and 10 blocks of OFF were taken for analysis, and the unit of design was SCANS. For both studies, the analyses performed were based on the whole brain.

### 2.7. Statistical Analysis

**Figure 2:**
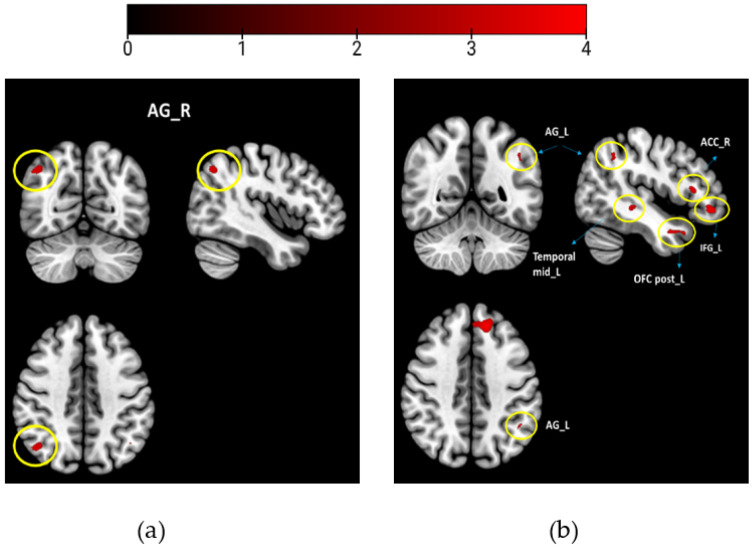
Between group results contrasting WB-associated odors > neutral odors (**a**) study 1; (**b**) study 2. Brain maps with thresholds set to *p*_uncorrected_ < 0.001, and a cluster size of k > 10 voxels. Encircled are the activation regions labeled. Colorbar presents the *t*-value.

Hypothesizing that odors with different WB associations would be processed differently, we combined and averaged the scans of the WB-associated odors (musk flower and orange for study 1) and compared them to the neutral odors (coffee and grass). Likewise, for study 2, we combined the WB-associated odors (vanilla and white soap) and compared them to the neutral odors (grass and sheets). We performed two sample *t*-tests to compare the odor groups, using the WB scores of individuals as covariates of interest for contrast: WB-associated odors vs. neutral odors (Figure 2). All activations were reported at *p*_uncorrected_ < 0.001 with acluster size of <10 voxels (study 1 and study 2).

For study 2, as there were 5 questionnaires with different score ranges, we normalized the distribution of scores per questionnaire. We then took the z scores of each and looked for significant differences between the questionnaires. No significant differences were found (F (4,200) = 2.31, *p* = 0.059). The averaged individual score for these 5 questionnaires and the mean centered valence scores were used as covariates during fMRI analysis when performing two sample *t*-tests for contrasts, WB-associated odors > neutral odors and WB-associated odors < neutral odors.

The statistical analysis was performed using IBM SPSS version 27 (SPSS Inc., Chicago, IL, USA). The significance level for all statistical tests was set to a *p* value of less than 0.05. 

## 3. Results

The intensity, pleasantness and WB ratings of the subjects recruited for fMRI scanning did not differ significantly (Table 1) from their pre-testing scores in both studies.

### fMRI Results

One sample *t*-test was conducted separately to look at the mean activation for WB-associated and neutral odors using valence as a covariate. The results can be found in the Supplementary Materials at the p family wise-error correction (FWE)-corrected threshold of <0.05. 

In the group analysis, the ratings and well-being questionnaire scores of the subjects were also used as covariates in the second level.

(1) WB-associated odors > neutral odors

study 1

When comparing brain activity for the WB-associated odors (musk flower and orange) to that for the neutral odors (coffee and grass), the right AG was activated (Table 2, Figure 2a). 

study 2

When comparing brain activity for the WB-associated odors (vanilla and white soap) to neutral odors (sheets and grass), we found activations of the left AG, left frontal superior medial, left inferior frontal gyrus, left rectus, right ACC, left posterior OFC, and left medial temporal lobe (Table 2, Figure 2b).

(2) WB-associated odors < neutral odors

In study 1 and study 2, no activations were found for the reverse contrast: WB-associated odors < neutral odors.

## 4. Discussion

In the present study, we aimed to evaluate the cerebral processing of odors with different degrees of WB association in two consecutive fMRI studies. In study 1, as an exploratory analysis, when comparing the two odor groups, we found enhanced activation in the right AG. Replicating the design in study 2, when evaluating the similar effect of WB-associated odors on a different population, we found activations in the left AG, left posterior OFC, inferior frontal and superior gyrus, right ACC, and left gyrus rectus.

In both studies, the consistent activation in the AG draws our attention, though lateralized differences exist. It is considered a high-level association area in the human brain [28] where multisensory information is combined and integrated to understand and give meaning to the stimuli. Structurally, the AG is an important intermediate that links and conveys information between different modalities and processing sub-systems [29]. In the present studies, it could be interpreted that subjects, when perceiving the WB-associated odors, showed the involvement of the AG, which shows its involvement in the attentional processing of new or alerting stimuli present in the environment [30]. This was also reported by [31], where authors observed high connectivity between the right AG and left mPFC for the contrast pleasant odors > no odor. The consistent activation of the AG in two different samples might explain its crucial role in emotional regulation as well as in cognitive and behavioral modulation by odorous stimuli [29,32]. From our results, we may predict that, when perceiving WB-associated odors, the AG plays the role of contextual integration, linking experiences and memories to the odors. To summarize, according to its functional aspects and neuroanatomy, the AG acts as a multimodal convergence hub, supporting attentional, episodic memory, semantic, and social cognitive processes [29,33] in response to presented stimuli.

In study 2, there were additional enhanced activations in the frontal brain parts and in the olfactory processing regions, the left posterior OFC. The extended activation of the OFC and of the inferior and medial part of the frontal gyrus along with the gyrus rectus points towards odor identification and olfactory memory retrieval [16]. Activation of the superior frontal gyrus may also reflect the retrieval of semantic information, as the region is functionally related to odor familiarity evaluation [34]. The temporal gyrus is a critical structure involved in social recognition; indeed, lesions to the temporal gyrus exhibit striking social and emotional problems [35]. Research suggests a role of the gyrus rectus in higher cognitive function, which therefore links it to personality. Being a subregion of the OFC, it relates to social functioning and cognitive empathy. In general, functions related to social cognition are related to the activation of the gyrus rectus.

The activation of the ACC, another region of olfactory processing, has an important role in projecting the information from the OFC to the limbic system [36]. The activation of these regions during the presentation of WB-associated odors may relate to the integration of emotions into cognition and decision-making [37].

It is worth noting that previous research evaluated the effects of two odors that are similar to those studied here as WB-associated odors, orange [38] and vanilla [39] in the context of contrasting food-related and non-food-related odors of comparable pleasantness. Both studies indicated that food odors could activate the “dopaminergic reward circuit”, and in the second study, the right anterior cingulate cortex was activated, as was found in the present study. A key difference of this study to previous work is that non-food-related odors, musk flower and white soap, were included as WB-associated odors. It is a notable finding that non-food-related odors can elicit such positive emotional associations, which is perhaps linked to the positive everyday context in which they are experienced.

According to our interpretation, differences in the results between the two studies exist due to two reasons. Firstly, in study 2, we used a different approach to study 1 to assess the WB state of an individual (during pre-testing). Secondly, overcoming the limitation of short odor exposure reported by the subjects in study 1, we focused on a longer exposure to the odors by giving the participants nose clips to wear at home. As WB can be better evaluated over a period rather than an instance, we made sure that subjects had a better idea of whether the odors would improve their WB or not. Longer exposure to the odors might have been the reason for the better activation of the social cognitive and emotional processing regions in study 2.

Whilst odors have a long cultural history of being used for WB-related therapies, it is not clear which brain regions are responsible for processing odors related to WB; more studies are needed to fully understand this pathway. Concerning these studies, the subject design could be a possible explanation of our uncorrected fMRI results, since we combined data for the two odors associated with WB and two neutral odors together in a two-sample *t*-test, which essentially means we averaged the activations in the same subjects which lead to lower signal strength. However, the results from one-sample *t*-test for each odor group showed FWE corrected results demonstrating good signal quality (Supplementary Materials). Although the results are uncorrected in nature, they in no way suggest an issue with the data quality as *p*_uncorrected_ values of <0.001 are symbolic of type II errors and are generally acceptable. The authors of [40] suggested that a *p* value of <0.005 at a cluster size of ‘K’ > 10 voxels has an equivalent false positive rate to FDR < 0.05. The complexity of neuroimaging analyses suggests that a variety of standards might be appropriate in different contexts.

## 5. Conclusions

To conclude, the present results indicate a link between the olfactory modality and higher order cognitive regions involved in emotions, memory, attentional processing and in learning, therefore adding meaning, value and context to the odors presented. The AG has not been much explored in the field of olfaction, particularly when it comes to WB. More research is needed to focus on its role in altering the emotional state of individuals when perceiving odors associated with WB.

## Figures and Tables

**Table 1:** Odor ratings in studies 1 and 2. Data are represented as means ± standard deviations; n.s. = non-significant.

Ratings (Range of Scale)	WB-Associated Odors	Neutral Odors	
study 1	musk flower + orange	coffee + grass	*p*-value
Intensity (0 to 10)	6.79 ± 1.98	6.62 ± 2.07	n.s.
Valence (−5 to +5)	3.00 ± 1.72	−0.42 ± 2.49	<0.001
Well-being (0 to 10)	7.25 ± 1.78	4.63 ± 2.15	<0.001
study 2	vanilla + white soap	sheets + grass	
Intensity (0 to 10)	6.59 ± 1.84	7.12 ± 1.87	n.s.
Valence (−5 to +5)	3.21 ± 1.85	−0.18 ± 2.69	<0.001
Well-being (0 to 10)	6.72 ± 2.11	3.4 ± 2.13	<0.001

**Table 2:** Regions activated for WB-associated contrast odors > neutral odors for study 1 and 2. Effect seen at *p*_uncorrected_ < 0.001 and a cluster size of k > 10 voxels; MNI coordinates are presented in x, y, and z; L, left hemisphere; R right hemisphere.

Study 1	k	T Value	x y z	Region
WB-associated odors > neutral odors	23	3.40	46 −68 44	Angular gyrus R
WB-associated odors < neutral odors	-	-	-	-
**study 2**				
WB-associated odors > neutral odors	111	4.51	−52 6 −26	Mid temporal L
	303	4.35	−10 50 24	Frontal superior medial L
	194	4.27	−14 38 40	Frontal superior L
	45	4.11	−8 24 −24	Gyrus Rectus L
	141	4.07	−42 42 −8	Inferior frontal gyrus L
	22	3.94	60 −4 −26	
	80	3.90	−52 24 10	Inferior frontal gyrus L
	76	3.78	−60 −16 −16	Mid temporal L
	58	3.66	14 38 4	Anterior cingulate R
	32	3.65	−34 30 −18	Posterior OFC L
	13	3.57	−46 −54 44	Angular gyrus L
WB-associated odors < neutral odors	-	-	-	-

## Data Availability

The data generated and analyzed are not publicly available due to the subjects’ confidentiality. They will be available from the corresponding author upon request.

## Supplementary Materials

The following supporting information can be downloaded at: https://www.mdpi.com/article/10.3390/brainsci13040576/s1, Supplementary data consists of results for the within group analysis for studies 1 and 2.
