# Supplementary material for: Neural Processing of Odors with Different Well-Being Associations—Findings from Two Consecutive Neuroimaging Studies
Source: Brain Sci. 2023 Mar 29;13(4):576. doi: 10.3390/brainsci13040576 (PMC10136803; doi:10.3390/brainsci13040576)
Supplement: Supplementary file 1 [file brainsci-13-00576-s001.zip › brainsci-2290564-supplementary.pdf]

<u>Supplementary data</u>

Study 1

WB associated odors

One sample t-test was done to look at the mean activation for WB associated odors (flower + orange), using valence score as covariate. Contrast chosen was odor (ON) > odorless air (OFF). Results reported at p FWE < 0.05. All results reported at whole brain level.

Left anterior prefrontal cortex

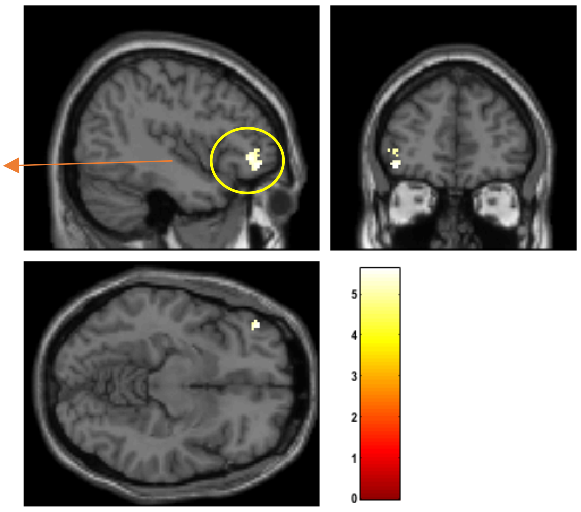

k	T value	x y z	region
116	5.62	-42 46 -10	Left anterior prefrontal cortex

Neutral odors

One sample t-test was done to look at the mean activation for neutral odors (coffee + grass), using valence score as covariate. Contrast chosen was odor (ON) > odorless air (OFF). Results reported at p FWE < 0.05.

k	T value	x y z	region
-	-	-	-

Study 2

WB associated odors

One sample t-test was done to look at the mean activation for WB associated odors (vanilla + soap), using valence score as covariate. Contrast chosen was odor (ON) > odorless air (OFF). Results reported at p FWE < 0.05.

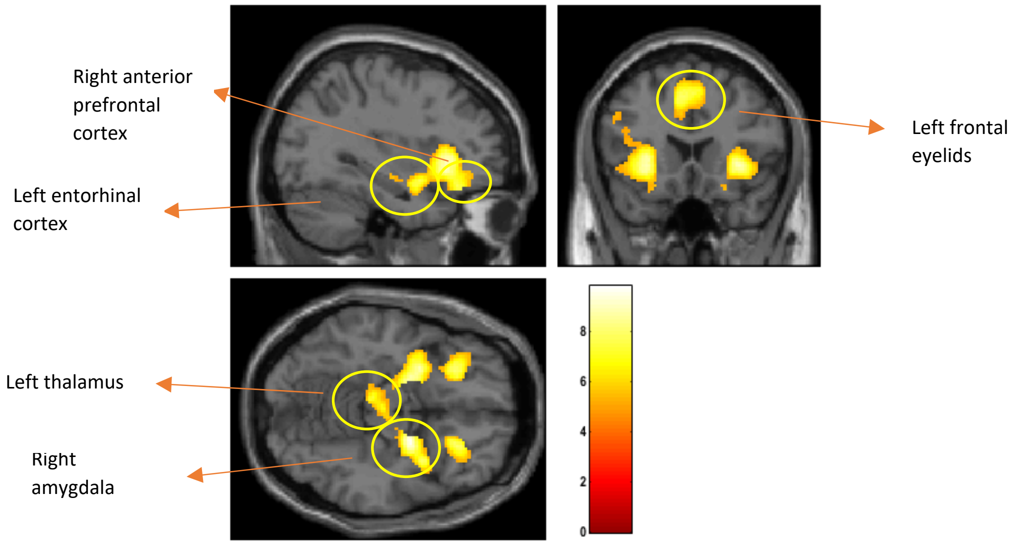

k	T value	x y z	region
3629	9.77	-26 2 -18	Left dorEntorhinal
2567	9.45	24 -2 -14	Right amygdala
1128	8.15	-6 16 50	Left frontal Eyefields
236	6.32	-2 -14 6	Left thalamus
21	5.42	46 48 12	Right ant prefrontal cortex

Neutral odors

One sample t-test was done to look at the mean activation for neutral odors (sheets + grass), using valence score as covariate. Contrast chosen was odor (ON) > odorless air (OFF). Results reported at p FWE < 0.05.

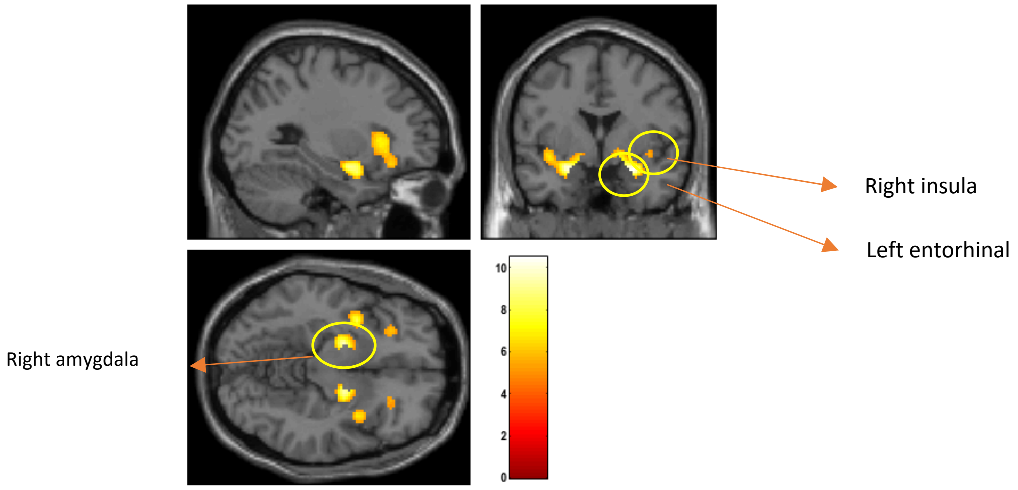

k	T value	x y z	region
510	10.50	26 2 -18	Left dorEntorhinal
1154	10.12	-24 2 -18	Right amygdala
507	7.99	32 26 0	Right insula
32	5.42	-6 16 52	Left frontal Eyelids
